# Impact of perioperative blood transfusion on long-term survival in patients with different stages of perihilar cholangiocarcinoma treated with curative resection: *A multicentre propensity score matching study*

**DOI:** 10.3389/fonc.2022.1059581

**Published:** 2022-10-31

**Authors:** Zhi-Peng Liu, Zheng-Jun Cheng, Hai-Su Dai, Shi-Yun Zhong, Dong-Chu Zhao, Yi Gong, Jing-Hua Zuo, Xiao-Yu Che, Wei-Yue Chen, Zi-Ran Wang, Ting Yu, Jun-Jie Cheng, Xing-Chao Liu, Jie Bai, Yan Jiang, Yan-Qi Zhang, Wan Yee Lau, Shi-Quan Deng, Zhi-Yu Chen

**Affiliations:** ^1^ Department of Hepatobiliary Surgery, Southwest Hospital, Third Military Medical University (Army Medical University), Chongqing, China; ^2^ Department of Hepatobiliary Surgery, Jiulongpo District Second People’s Hospital, Chongqing, China; ^3^ Clinical Research Center of Oncology, Lishui Hospital of Zhejiang University, Lishui, China; ^4^ Department of General Surgery, 903rd Hospital of People’s Liberation Army, Hangzhou, China; ^5^ Department of Hepatobiliary Surgery, Sichuan Provincial People’s Hospital, Chengdu, China; ^6^ Department of Health Statistics, College of Military Preventive Medicine, Third Military Medical University (Army Medical University), Chongqing, China; ^7^ Faculty of Medicine, The Chinese University of Hong Kong, Hong Kong, Hong Kong SAR, China; ^8^ Department of Hepatobiliary Surgery, Chongqing Jiulongpo District Integrated Traditional Chinese and Western Medicine Hospital, Chongqing, Hong Kong SAR, China

**Keywords:** perihilar cholangiocarcinoma, perioperative blood transfusion, resection, survival, recurrence

## Abstract

**Background & aim:**

The association of perioperative blood transfusion (PBT) with long-term survival in perihilar cholangiocarcinoma (pCCA) patients after surgical resection with curative intent is controversial and may differ among different stages of the disease. This study aimed to investigate the impact of PBT on long-term survival of patients with different stages of pCCA.

**Methods:**

Consecutive pCCA patients from three hospitals treated with curative resection from 2012 to 2019 were enrolled and divided into the PBT and non-PBT groups. Propensity score matching (PSM) was used to balance differences in baseline characteristics between the PBT and non-PBT groups. Kaplan–Meier curves and log-rank test were used to compare overall survival (OS) and recurrence-free survival (RFS) between patients with all tumor stages, early stage (8th AJCC stage I), and non-early stage (8th AJCC stage II-IV) pCCA in the PBT and non-PBT groups. Cox regression analysis was used to determine the impact of PBT on OS and RFS of these patients.

**Results:**

302 pCCA patients treated with curative resection were enrolled into this study. Before PSM, 68 patients (22 patients in the PBT group) were in the early stage and 234 patients (108 patients in the PBT group) were in the non-early stage. Patients with early stage pCCA in the PBT group had significantly lower OS and RFS rates than those in the non-PBT group. However, there were with no significant differences between the 2 groups with all tumor stages and non-early stage pCCA. After PSM, there were 18 matched pairs of patients with early stage and 72 matched pairs of patients with non-early stage. Similar results were obtained in the pre- and post-PSM cohorts: patients with early stage pCCA in the PBT group showed significantly lower OS and RFS rates than those in the non-PBT group, but there were no significant differences between the 2 groups for patients with all tumor stages and non-early stage pCCA. Cox regression analysis demonstrated that PBT was independently associated with worse OS and RFS for patients with early stage pCCA.

**Conclusions:**

PBT had a negative impact on long-term survival in patients with early stage pCCA after curative resection, but not in patients with non-early stage pCCA.

## Introduction

Cholangiocarcinoma accounts for 3% of all gastrointestinal tumors and represents 10~25% of all primary hepatic malignancies globally (1, 2). Perihilar cholangiocarcinoma (pCCA) is the most common type of cholangiocarcinoma, accounting for approximately 60% of these cases (3). The only treatment that can result in long-term survival for patients with pCCA is curative resection (4, 5). However, the complicated nature of the surgical procedure which includes bile duct resection and reconstruction, hepatectomy, perihilar dissection, vascular resection and reconstruction if necessary, as well as coagulopathy due to preoperative jaundice, make the possibility of intraoperative bleeding and perioperative blood transfusion extremely likely (6).

Perioperative blood transfusion (PBT) plays an essential role in perioperative safety of pCCA patients. However, the impact of PBT on long-term survival in pCCA patients treated with curative resection has been controversial. Müller et al. indicated that allogeneic blood transfusion did not affect long-term survival after curative resection for advanced cholangiocarcinoma (7). However, Kimura et al. indicated that PBT was a poor prognostic factor for hilar cholangiocarcinoma treated with curative resection (8). Both these two studies focused on long-term survival in cholangiocarcinoma patients following curative resection**,** they reached completely different conclusions. In fact, allogeneic blood transfusion has been demonstrated to have immunosuppressive effects, which are associated with a higher chance of tumor recurrence and a poor long-term prognosis in patients with malignancies (9, 10). There are two possible explanations for the different results obtained in the above two mentioned studies. First, both these studies were single-centre studies with small sample sizes, and the results were of low-level of medical evidence. Second, the conclusions drawn based on the total cohort did not apply to an individual, as the patients had tumors of different stages. Previous studies on hepatocellular carcinoma showed PBT to have different impact on long-term survival in different tumor stages (11, 12). However, the impact of PBT on long-term survival has not been studied in patients with different stages of pCCA.

Ethical reasons do not allow clinical researchers to conduct a randomized controlled trial on PBT. To improve the level of medical evidence, 302 patients from 3 institutions were identified from a multicentre database to be included to conduct this first study by using propensity score matching (PSM) analysis to study the impact of PBT on long-term survival in patients with different stages of pCCA treated with curative resection.

## Methods

### Patients

From February 2012 to February 2019, consecutive pCCA patients treated with curative resection at three hospitals (Southwest Hospital, Sichuan Provincial People’s Hospital, Jiulongpo District Second People’s Hospital) were enrolled in this study. Tumors originating from common hepatic duct, junction of common hepatic duct, and left/right first-order hepatic ducts were all grouped as pCCA. All diagnoses were confirmed by postoperative histopathology. The exclusion criteria were patients with (1): recurrent pCCA; (2) loss to follow-up; (3) lack of data for essential variables; and (4) death within 30 days after curative resection. This study complied with the Declaration of Helsinki and was approved by the Ethics Committees of the 3 participating hospitals. Due to its retrospective nature and because all data were deidentified, informed consent was exempted.

### Surgical procedure

Curative resection was defined as resection resulting in microscopically clear margins. Curative resection included bile duct resection, biliary reconstruction, hepatectomy, lymph node dissection, and vascular reconstruction for vascular invasion as previously reported (13–15). Curative resection was performed by experienced surgeons in hepatobiliary surgery in the 3 institutions.

### Data collection

Data was prospectively collected into a database used by the 3 participating hospitals and the study was conducted retrospectively. The data collected on patient demographic, preoperative laboratory, postoperative histopathological and surgical variables included gender, age, comorbidity, preoperative jaundice, preoperative hepatolithiasis, chronic hepatitis, American Society of Anesthesiologists (ASA) score, alanine aminotransferase (ALT), aspartate transaminase (AST), international normalized ratio (INR), albumin (ALB), hemoglobin (HGB), carbohydrate antigen 19-9 (CA 19-9), tumor size, degree of tumor differentiation, macrovascular invasion, microvascular invasion, lymph node (LN) involvement, nerve invasion, cirrhosis, 8th American Joint Committee on Cancer (AJCC) staging (16), extent of hepatectomy, PBT, perioperative blood loss and operation time.

Patients were divided into two groups according to the upper or lower limits of normal of each preoperative laboratory variable. Specifically, the following thresholds were employed: ALT and AST: 40 U/L, INR: 1.15, ALB: 35 g/L, HGB: 120 g/L, and CA 19-9: 37 U/L (13, 14, 17). All postoperative histopathological variables were confirmed by postoperative histopathological examination of tumor or nontumor tissues. Preoperative jaundice was defined as a preoperative total bilirubin higher than 37 μmol/L. Extent of hepatectomy was divided into major hepatectomy (three or more resected Couinaud liver segments) and minor hepatectomy (two or less resected Couinaud liver segments). In previous studies, pCCA patients with a tumor size > 3 cm showed poor long-term survival (13, 14). As a consequence, 3 cm was used to divide patients into 2 groups. Both portal vein invasion and hepatic artery invasion were considered as macrovascular invasion.

### Perioperative blood transfusion

PBT was defined as transfusion of whole blood and/or packed red blood cells (PRBCs) either during surgery or within 7 days of surgery as determined from the surgical and postoperative medical records. PBT excluded autologous blood, allogeneic platelets, fresh frozen plasma, and cryoprecipitate. The need for intraoperative blood transfusions was determined by excessive intraoperative blood loss and/or hemodynamic instability. Postoperative blood transfusions were administered if the patient’s hemoglobin level was below 70 g/L or the patient was hemodynamically unstable. Two units were the standard for transfusion (one unit of PRBCs refers to the red blood cells isolated from 200 ml of whole blood).

### Survival outcomes and follow-up

The main outcomes were overall survival (OS) and recurrence-free survival (RFS). OS was defined as the interval from curative resection to death or the last follow-up. The definition of RFS for patients with recurrence was the interval from curative resection to recurrence, and for patients with no recurrence as the interval from curative resection to death or last follow-up. This study was censored on February 28, 2022. After discharged from hospital, patients were followed-up once every 1-2 months for 2 years after curative resection, once every 3-4 months for 3-5 years and then once every 6 months for 5 years. Contrast-enhanced ultrasonography, contrast-enhanced computed tomography, and/or magnetic resonance cholangiopancreatography were performed at each follow-up. Conservative therapy, systemic chemotherapy, or repeat surgical resection were performed if patients were confirmed to have relapsed.

### Statistical analysis

Continuous variables with normal distributions were presented as means and standard deviations (SDs) and were compared using the Student’s t test, whereas continuous variables with non-normal distributions weare presented as medians with interquartile ranges (IQRs) and were compared using the Mann−Whitney U test. Categorical variables were presented as frequencies and percentages and were compared using the Pearson’s chi-square test. All patients were divided into two groups according to whether PBT was given. All the baseline characteristics of the two groups were compared. To overcome the influence of selection bias, PSM was used to balance the differences in the baseline characteristics between the PBT and non-PBT groups. Tendency scoring system was used for PSM to integrate all observed variable information, in order to balance variable and reduce the bias. Potential variables which might affect PBT were included into the propensity model, including preoperative jaundice, ASA grade, INR, ALB, HGB, tumor size, cirrhosis, and extent of hepatectomy. Propensity scores for pCCA patients who received PBT or not were created using logistic regression estimation. A one-to-one match between the two groups was then performed using the nearest-neighbor matching method with a caliper width equal to 0.2 of the standard deviation of the logit of the propensity score. Kaplan–Meier curves were used to calculate the OS and RFS rates of patients, and the log-rank test was used for comparisons. Variables with a significance level of *P* < 0.1 in univariate analysis were included in multivariate analysis using the Cox regression model to determine independent predictors of OS and RFS. In addition, using the 8th AJCC staging system, all patients were divided into the early stage (AJCC stage I) group and the non-early stage (AJCC stage II-IV) group. Subgroup analysis was used to investigate the impact of PBT on OS and RFS for patients with different tumor stagings. SPSS^®^ version 26.0 (IBM, Armonk, New York, United States) was used for all statistical analyses. A *P* value (two-sided) < 0.05 was considered statistically significant.

## Results

### Characteristics of all pCCA patients

Of 364 pCCA patients treated with curative resection during the study period, 62 patients were excluded according to the exclusion criteria, resulting in 302 pCCA patients being included in this study (**Supplement Figure 1**). There were 198 (65.6%) males, and 125 (41.4%) patients were more than 60 years old. The median follow-up time was 22.5 months. The PBT group had 130 patients (43.0%), and the non-PBT group had 172 patients (57.0%). Before PSM, baseline characteristics showed the PBT group to have significantly more patients with preoperative jaundice, ASA grade > II, INR > 1.15, ALB < 35 g/L, HGB < 120 g/L, tumor size > 3 cm, 8th AJCC stage II-IV disease, major hepatectomy, blood loss > 500 mL and operation time > 360 min than the non-PBT group. After PSM with 90 matched pairs of patients were analyzed, baseline characteristics of the PBT group still showed significantly more patients with the 8th AJCC stage II-IV disease than the non-PBT group (**Table 1**).

**Table 1:** Clinicopathologic characteristics of the PBT and non-PBT groups among all pCCA patients treated with curative resection.

Variables	Before PSM			After PSM		
	PBT (n=130)	Non-PBT (n=172)	*P* value ^a^	PBT (n=90)	Non-PBT (n=90)	*P* value ^a^
Male	90 (69.2)	108 (62.8)	0.224	62 (68.2)	62 (68.2)	1.000
Age > 60 years	56 (43.1)	69 (40.1)	0.605	40 (44.4)	46 (51.1)	0.371
Comorbidity	33 (25.4)	39 (22.7)	0.584	27 (30.0)	21 (23.3)	0.312
Preoperative jaundice	108 (83.1)	102 (59.3)	< 0.001	70 (77.8)	70 (77.8)	1.000
Preoperative hepatolithiasis	11 (8.5)	14 (8.1)	0.920	8 (8.9)	7 (7.8)	0.787
Chronic hepatitis	16 (12.3)	11 (6.4)	0.075	10 (11.1)	9 (10.0)	0.808
ASA grade > II	19 (14.6)	12 (7.0)	0.030	9 (10.0)	10 (11.1)	0.808
ALT > 40 U/L	110 (84.6)	140 (81.4)	0.463	76 (84.4)	76 (84.4)	1.000
AST > 40 U/L	109 (83.8)	135 (78.5)	0.242	76 (84.4)	75 (83.3)	0.839
INR > 1.15	17 (13.1)	10 (5.8)	0.029	5 (5.6)	8 (8.9)	0.388
ALB < 35 g/L	59 (45.4)	51 (29.7)	0.005	40 (44.4)	34 (37.8)	0.363
HGB < 120 g/L	40 (30.8)	33 (19.2)	0.020	27 (30.0)	22 (24.4)	0.402
CA 19-9 > 37 U/L	98 (75.4)	126 (73.3)	0.676	66 (73.3)	65 (72.2)	0.867
Tumor size > 3 cm	59 (45.4)	56 (32.6)	0.023	39 (43.3)	40 (44.4)	0.881
Poor differentiation	22 (16.9)	20 (11.6)	0.188	16 (17.8)	13 (14.4)	0.543
Macrovascular invasion	39 (30.0)	43 (25.0)	0.333	20 (22.2)	12 (13.3)	0.119
Microvascular invasion	15 (11.5)	19 (11.0)	0.893	8 (8.9)	11 (12.2)	0.467
LN involvement	55 (42.3)	61 (35.5)	0.226	35 (38.9)	33 (36.7)	0.758
Peripheral nerve invasion	46 (35.4)	53 (30.8)	0.402	34 (37.8)	29 (32.2)	0.435
Cirrhosis	15 (11.5)	10 (5.8)	0.074	6 (6.7)	9 (10.0)	0.418
8th AJCC stage II-IV	108 (83.1)	126 (73.3)	0.043	75 (83.3)	56 (62.2)	0.001
Major hepatectomy	94 (72.3)	102 (59.3)	0.019	62 (68.9)	59 (65.6)	0.634
Blood loss > 500 mL	98 (75.4)	99 (57.6)	0.001	63 (70.0)	51 (56.7)	0.063
Operation time > 360 min	76 (58.5)	75 (43.6)	0.011	45 (50.0)	41 (45.6)	0.551

a The calibration formula of chi-square test was used.

ALB, albumin; ALT, alanine aminotransferase; AJCC, American Joint Committee on Cancer; ASA, American Society of Anesthesiologists; AST, aspartate transaminase; CA 19-9, carbohydrate antigen 19-9; HGB, hemoglobin; INR, international normalized ratio; LN, lymph node; pCCA, perihilar cholangiocarcinoma; PBT, perioperative blood transfusion; PSM, propensity score matching.

### Long-term survival of all pCCA patients

**Figure 1:**
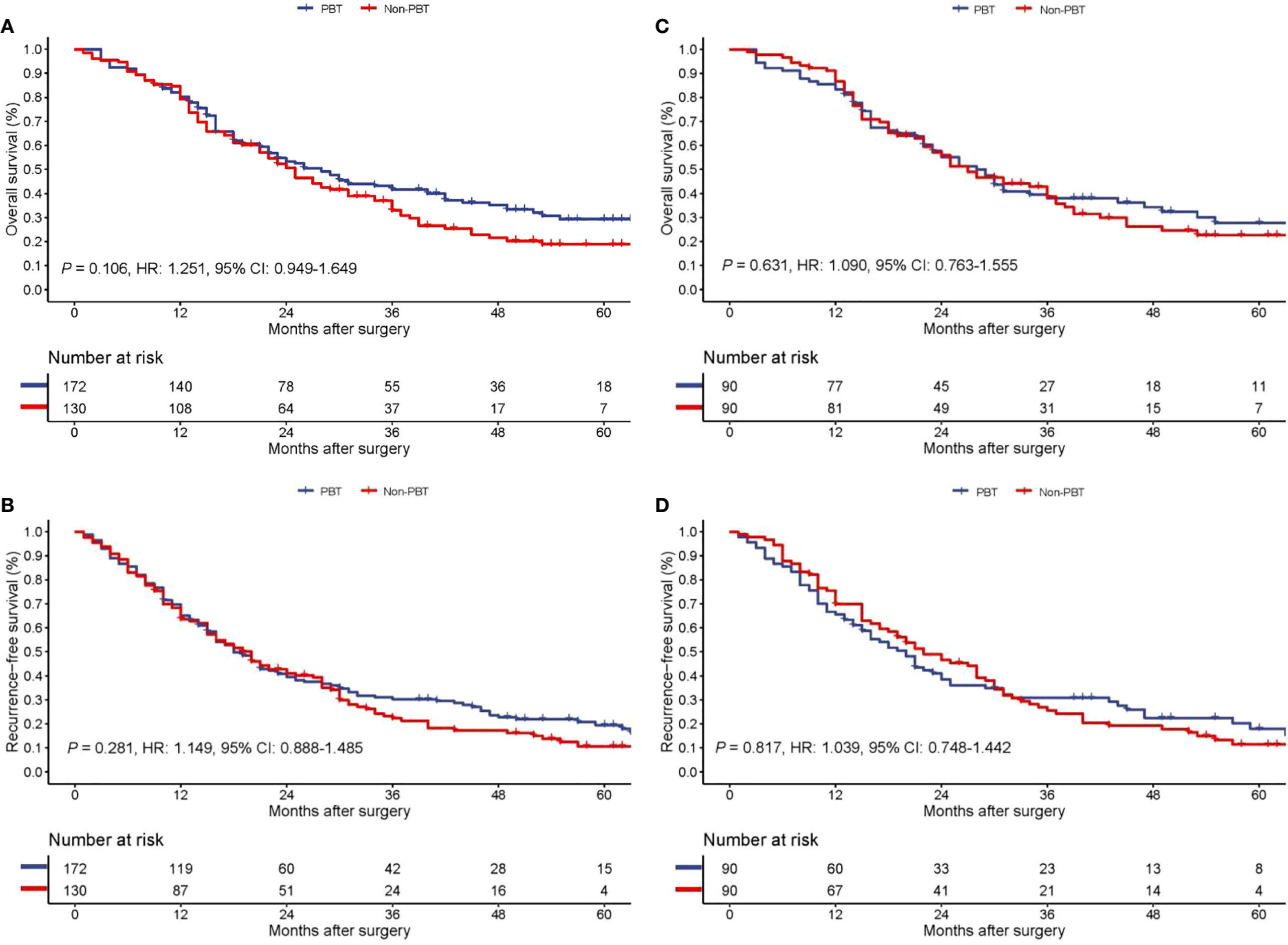
Kaplan−Meier curves of overall survival **(A)** and recurrence-free survival **(B)** between the PBT and non-PBT groups among all pCCA patients treated with curative resection before PSM. Kaplan−Meier curves of overall survival **(C)** and recurrence-free survival **(D)** between the PBT and non-PBT groups among all pCCA patients treated with curative resection after PSM. CI, confidence interval; HR, hazard ratio; PBT, perioperative blood transfusion; PSM, propensity score matching.

On follow-up, before PSM, the 5-year OS rates for all pCCA patients treated with curative resection were 18.9% in the PBT group and 29.4% in the non-PBT group, respectively, while the 5-year RFS rates were 10.6% in the PBT group and 19.5% in the non-PBT group, respectively. After PSM, the 5-year OS rates for all pCCA patients treated with curative resection were 22.7% in the PBT group and 27.8% in the non-PBT group, respectively, while the 5-year RFS rates were 11.4% in the PBT group and 18.0% in the non-PBT group, respectively (**Supplement Table 1**). Both before and after PSM, Kaplan–Meier curves revealed that there were no significant differences between the PBT and non-PBT groups in OS and RFS (**Figure 1**).

### Characteristics of patients with early stage pCCA

68 patients with early stage (AJCC stage I) pCCA were treated with curative resection. Among these patients, 22 patients (32.4%) were in the PBT group, and 46 patients (67.6%) were in the non-PBT group. Before PSM, baseline characteristics showed the PBT group to have significantly more patients with an ALB < 35 g/L, HGB < 120 g/L and blood loss > 500 mL than the non-PBT group. After PSM with 18 matched pairs of patients being analyzed, there were no significant differences in baseline characteristics between the PBT group and the non-PBT group (**Table 2**).

**Table 2:** Clinicopathologic characteristics of the PBT and non-PBT groups among patients with early stage (8th AJCC stage I) pCCA treated with curative resection.

Variables	Before PSM			After PSM		
	PBT (n = 22)	Non-PBT (n = 46)	*P* value ^a^	PBT (n = 18)	Non-PBT (n = 18)	*P* value
Male	16 (72.7)	35 (76.1)	0.765	15 (83.3)	12 (66.7)	0.248
Age > 60 years	15 (68.2)	28 (60.9)	0.559	13 (72.3)	13 (72.3)	1.000
Comorbidity	8 (36.4)	9 (19.6)	0.134	6 (33.3)	6 (33.3)	1.000
Preoperative jaundice	18 (81.8)	27 (58.7)	0.059	14 (77.8)	15 (83.3)	0.674
Preoperative hepatolithiasis	1 (4.5)	4 (8.7)	0.540	1 (5.6)	1 (5.6)	1.000
Chronic hepatitis	3 (13.6)	5 (10.9)	0.740	3 (16.7)	2 (11.1)	0.630
ASA grade > II	3 (13.6)	6 (13.0)	0.946	3 (16.7)	3 (16.7)	1.000
ALT > 40 U/L	19 (86.4)	37 (80.4)	0.549	17 (94.4)	15 (83.3)	0.289
AST > 40 U/L	17 (77.3)	36 (78.3)	0.927	15 (83.3)	15 (83.3)	1.000
INR > 1.15	4 (18.2)	4 (8.7)	0.256	3 (16.7)	1 (5.6)	0.289
ALB < 35 g/L	11 (50.0)	11 (23.9)	0.031	7 (38.9)	7 (38.9)	1.000
HGB < 120 g/L	11 (50.0)	8 (17.4)	0.005	7 (38.9)	6 (33.3)	0.729
CA 19-9 > 37 U/L	14 (63.6)	23 (50.0)	0.291	11 (61.1)	10 (55.6)	0.735
Tumor size > 3 cm	7 (31.8)	11 (23.9)	0.489	7 (38.9)	5 (27.8)	0.480
Poor differentiation	2 (9.1)	3 (6.5)	0.704	2 (11.1)	2 (11.1)	1.000
Cirrhosis	4 (18.2)	4 (8.7)	0.256	4 (22.2)	2 (11.1)	0.371
Major hepatectomy	13 (59.1)	18 (39.1)	0.122	12 (66.7)	7 (38.9)	0.095
Blood loss > 500 mL	17 (77.3)	23 (50.0)	0.003	13 (72.2)	9 (50.0)	0.171
Operation time > 360 min	9 (40.9)	12 (26.1)	0.216	7 (38.9)	4 (22.2)	0.278

a The calibration formula of chi-square test was used.

ALB, albumin; ALT, alanine aminotransferase; AJCC, American Joint Committee on Cancer; ASA, American Society of Anesthesiologists; AST, aspartate transaminase; CA 19-9, carbohydrate antigen 19-9; HGB, hemoglobin; INR, international normalized ratio; pCCA, perihilar cholangiocarcinoma; PBT, perioperative blood transfusion; PSM, propensity score matching.

### Long-term survival of patients with early stage pCCA

**Figure 2:**
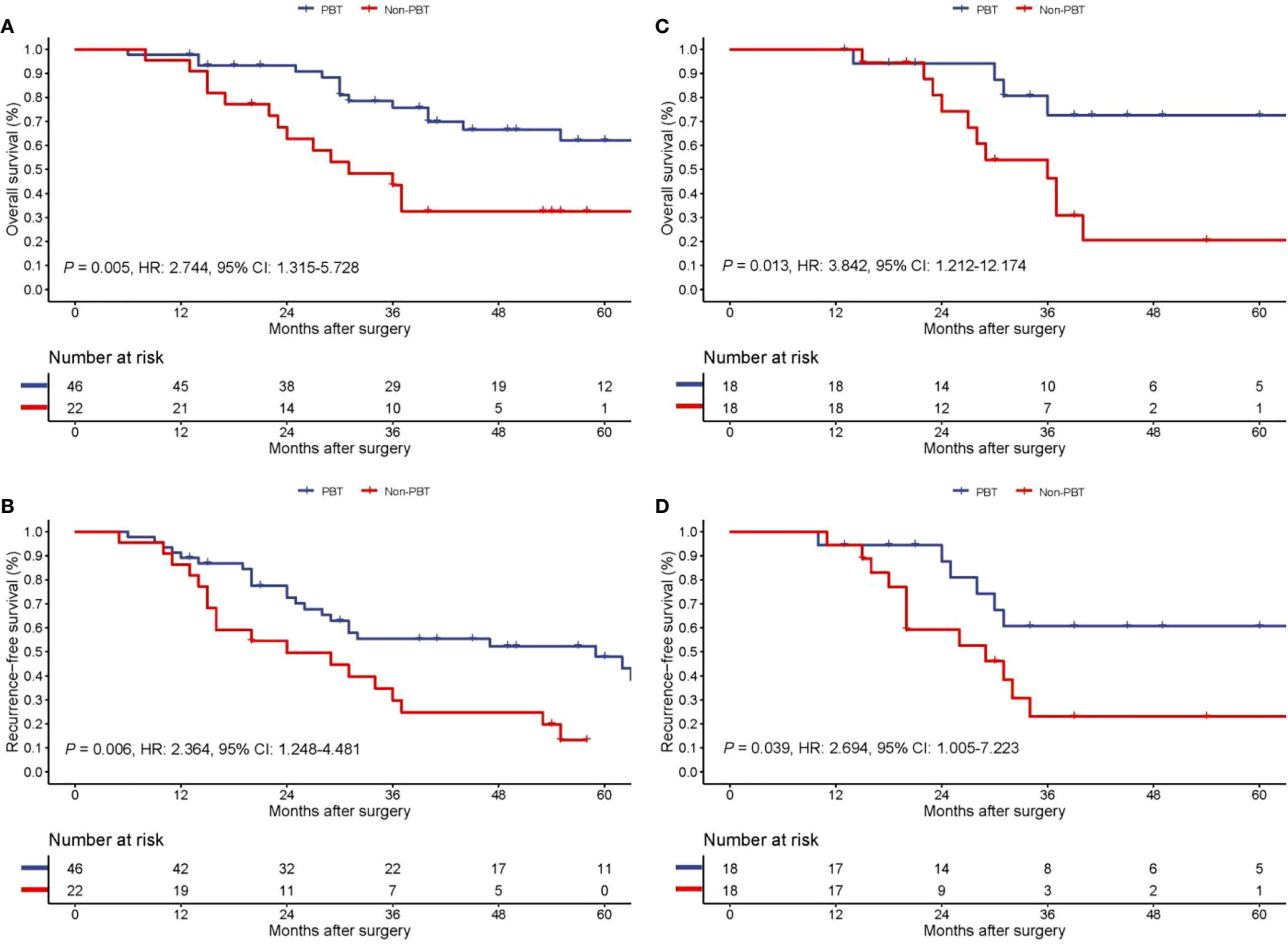
Kaplan−Meier curves of overall survival **(A)** and recurrence-free survival **(B)** between the PBT and non-PBT group among patients with early stage (8th AJCC stage I) pCCA treated with curative resection before PSM. Kaplan−Meier curves of overall survival **(C)** and recurrence-free survival **(D)** between the PBT and non-PBT group among patients with early stage (8th AJCC stage I) pCCA treated with curative resection after PSM. CI, confidence interval; HR, hazard ratio; PBT, perioperative blood transfusion; PSM, propensity score matching.

On follow-up, before PSM, the 5-year OS rates of patients with early stage pCCA treated with curative resection were 32.6% in the PBT group and 62.2% in the non-PBT group, respectively, while the 5-year RFS rates were 13.2% in the PBT group and 47.9% in the non-PBT group, respectively. After PSM, the 5-year OS rates of patients with early stage pCCA treated with curative resection were 20.6% in the PBT group and 72.6% in the non-PBT group, respectively, while the 5-year RFS rates were 23.0% in the PBT group and 60.7% in the non-PBT group, respectively (**Supplement Table 2**). Both before and after PSM, Kaplan–Meier curves revealed that in patients with early stage pCCA, the OS and RFS rates in the PBT group were significantly lower than those in the non-PBT group (**Figure 2**). After PSM, multivariable analyses revealed that for patients with early stage pCCA, PBT and tumor size >3 cm to be independently associated with worse OS (**Table 3**) and RFS (**Table 4**).

**Table 3:** Univariable and multivariable analyses of independent risk factors for overall survival among patients with early stage (8th AJCC stage I) pCCA treated with curative resection after propensity score matching.

Variables	Comparison	Univariable analyses		Multivariable analyses*	
		*P* value	HR (95% CI)	*P* value	HR (95% CI)
PBT	Yes *vs.* No	0.022	3.842 (1.212-12.174)	0.024	3.772 (1.187-11.983)
Sex	Male *vs.* Female	0.863	0.904 (0.286-2.852)		
Age, years	> 60 vs. ≤ 60	0.981	1.014 (0.322-3.195)		
Comorbidity	Yes vs. No	0.445	1.497 (0.532-4.212)		
Preoperative jaundice	Yes vs. No	0.561	1.556 (0.350-6.918)		
Preoperative hepatolithiasis	Yes vs. No	0.560	1.841 (0.237-14.302)		
Chronic hepatitis	Yes vs. No	0.631	1.443 (0.323-6.438)		
ASA grade > II	Yes vs. No	0.358	2.032 (0.448-9.211)		
ALT, U/L	> 40 *vs.* ≤ 40	0.320	2.807 (0.367-21.453)		
AST, U/L	> 40 vs. ≤ 40	0.124	4.932 (0.645-37.704)		
INR	> 1.15 vs. ≤ 1.15	0.210	2.265 (0.630-8.146)		
ALB, g/L	< 35 vs. ≥ 35	0.572	1.349 (0.478-3.806)		
HGB, g/L	< 110 vs. ≥ 100	0.176	2.017 (0.731-5.569)		
CA 19-9, U/L	> 37 vs. ≤ 37	0.065	2.959 (0.937-9.350)	0.147	NA
Tumor size, cm	> 3 vs. ≤ 3	0.013	3.707 (1.317-10.432)	0.015	3.683 (1.287-10.279)
Poor differentiation	Yes vs. No	0.531	1.612 (0.362-7.178)		
Cirrhosis	Yes vs. No	0.483	1.574 (0.442-5.601)		
Major hepatectomy	Yes vs. No	0.146	2.346 (0.743-7.415)		

*Those variables that had a P <.10 in the univariable analyses were entered into the multivariable analyses.

ALB, albumin; ALT, alanine aminotransferase; AJCC, American Joint Committee on Cancer; ASA, American Society of Anesthesiologists; AST, aspartate transaminase; CA 19-9, carbohydrate antigen 19-9; HGB, hemoglobin; INR, international normalized ratio; NA, not available; pCCA, perihilar cholangiocarcinoma; PBT, perioperative blood transfusion.

**Table 4:** Univariable and multivariable analyses of independent risk factors for recurrence-free survival among patients with early stage (8th AJCC stage I) pCCA treated with curative resection after propensity score matching.

Variables	Comparison	Univariable analyses		Multivariable analyses*	
		*P* value	HR (95% CI)	*P* value	HR (95% CI)
PBT	Yes vs. No	0.049	2.694 (1.005-7.223)	0.015	3.709 (1.289-10.674)
Sex	Male vs. Female	0.724	0.829 (0.294-2.337)		
Age, years	> 60 vs. ≤ 60	0.568	1.383 (0.454-4.216)		
Comorbidity	Yes vs. No	0.323	1.615 (0.624-4.181)		
Preoperative jaundice	Yes vs. No	0.738	1.263 (0.358-4.274)		
Preoperative hepatolithiasis	Yes vs. No	0.855	1.207 (0.160-9.135)		
Chronic hepatitis	Yes vs. No	0.963	1.035 (0.237-4.519)		
ASA grade > II	Yes vs. No	0.646	1.419 (0.320-6.296)		
ALT, U/L	> 40 vs. ≤ 40	0.235	3.399 (0.451-25.635)		
AST, U/L	> 40 vs. ≤ 40	0.082	6.034 (0.798-45.615)	0.060	NA
INR	> 1.15 vs. ≤ 1.15	0.367	1.775 (0.511-6.167)		
ALB, g/L	< 35 vs. ≥ 35	0.727	1.184 (0.458-3.062)		
HGB, g/L	< 110 vs. ≥ 100	0.367	1.539 (0.603-3.933)		
CA 19-9, U/L	> 37 vs. ≤ 37	0.126	2.160 (0.805-5.794)		
Tumor size, cm	> 3 vs. ≤ 3	0.023	2.954 (1.160-7.524)	0.006	4.108 (1.485-11.362)
Poor differentiation	Yes vs. No	0.228	2.160 (0.617-7.558)		
Cirrhosis	Yes vs. No	0.885	1.096 (0.316-3.794)		
Major hepatectomy	Yes vs. No	0.573	1.314 (0.509-3.393)		

*Those variables that had a P <.10 in the univariable analyses were entered into the multivariable analyses.

ALB, albumin; ALT, alanine aminotransferase; AJCC, American Joint Committee on Cancer; ASA, American Society of Anesthesiologists; AST, aspartate transaminase; CA 19-9, carbohydrate antigen 19-9; HGB, hemoglobin; INR, international normalized ratio; NA, not available; pCCA, perihilar cholangiocarcinoma; PBT, perioperative blood transfusion.

### Characteristics of patients with non-early stage pCCA

234 patients with non-early stage (AJCC stage II-IV) pCCA were treated with curative resection. Of which, 108 patients with AJCC stage II, pCCA 103 patients with AJCC stage III pCCA, 23 patients with AJCC stage IV pCCA were treated with curative resection. The PBT group had 108 patients (46.2%), and the non-PBT group had 126 patients (53.8%). Before PSM, baseline characteristics showed the PBT group to have significantly more patients with preoperative jaundice, chronic hepatitis, ASA > II grade, INR > 1.15, ALB < 35 g/L, blood loss > 500 ml, and operation time > 360 min than the non-PBT group. After PSM, with 72 matched pairs of patients were analyzed, there were no significant differences in baseline characteristics between the PBT group and non-PBT group (**Table 5**).

**Table 5:** Clinicopathologic characteristics of the PBT and non-PBT groups among patients with non-early stage (8th AJCC stage II-IV) pCCA treated with curative resection.

Variables	Before PSM			After PSM		
	PBT (n = 108)	Non-PBT (n = 126)	*P* value ^a^	PBT (n = 72)	Non-PBT (n = 72)	*P* value ^a^
Male	74 (68.5)	73 (57.9)	0.096	48 (66.7)	41 (56.9)	0.230
Age > 60 years	41 (38.0)	41 (32.5)	0.387	27 (37.5)	25 (34.7)	0.729
Comorbidity	25 (23.1)	30 (23.8)	0.906	19 (26.4)	15 (20.8)	0.433
Preoperative jaundice	90 (83.3)	77 (61.1)	< 0.001	56 (77.8)	58 (80.6)	0.682
Preoperative hepatolithiasis	10 (9.3)	10 (7.9)	0.719	8 (11.1)	5 (6.9)	0.383
Chronic hepatitis	13 (12.0)	6 (4.8)	0.043	6 (8.3)	5 (6.9)	0.754
ASA grade > II	16 (14.8)	6 (4.8)	0.009	7 (9.7)	4 (5.6)	0.347
ALT > 40 U/L	91 (84.3)	103 (81.7)	0.611	64 (88.9)	59 (81.9)	0.238
AST > 40 U/L	92 (85.2)	99 (78.6)	0.194	62 (86.1)	56 (77.8)	0.194
INR > 1.15	13 (12.0)	6 (4.8)	0.043	4 (5.6)	4 (5.6)	1.000
ALB < 35 g/L	48 (44.4)	40 (31.7)	0.046	30 (41.7)	27 (37.5)	0.609
HGB < 120 g/L	29 (26.9)	25 (19.8)	0.205	20 (27.8)	11 (15.3)	0.068
CA 19-9 > 37 U/L	84 (77.8)	103 (81.7)	0.451	56 (77.8)	57 (79.2)	0.839
Tumor size > 3 cm	52 (48.1)	45 (35.7)	0.055	27 (37.5)	33 (45.8)	0.310
Poor differentiation	20 (18.5)	17 (13.5)	0.294	12 (16.7)	12 (16.7)	1.000
Macrovascular invasion	39 (36.1)	43 (34.1)	0.751	28 (38.9)	18 (25.0)	0.074
Microvascular invasion	15 (12.0)	19 (15.1)	0.797	6 (8.3)	12 (16.7)	0.131
LN involvement	55 (50.9)	61 (48.4)	0.701	30 (41.7)	38 (52.8)	0.182
Peripheral nerve invasion	46 (42.6)	53 (42.1)	0.935	26 (36.1)	24 (33.3)	0.726
Cirrhosis	39 (36.1)	43 (34.1)	0.752	7 (9.7)	2 (2.8)	0.085
Major hepatectomy	15 (13.9)	19 (15.1)	0.797	51 (70.8)	51 (70.8)	1.000
Blood loss > 500 mL	55 (50.9)	61 (48.4)	0.702	51 (70.8)	43 (59.7)	0.161
Operation time > 360 min	40 (37.0)	40 (31.7)	0.396	39 (54.2)	37 (51.4)	0.738

a The calibration formula of chi-square test was used.

ALB, albumin; ALT, alanine aminotransferase; AJCC, American Joint Committee on Cancer; ASA, American Society of Anesthesiologists; AST, aspartate transaminase; CA 19-9, carbohydrate antigen 19-9; HGB, hemoglobin; INR, international normalized ratio; pCCA, perihilar cholangiocarcinoma; PBT, perioperative blood transfusion; PSM, propensity score matching.

### Long-term survival for patients with non-early stage pCCA

**Figure 3:**
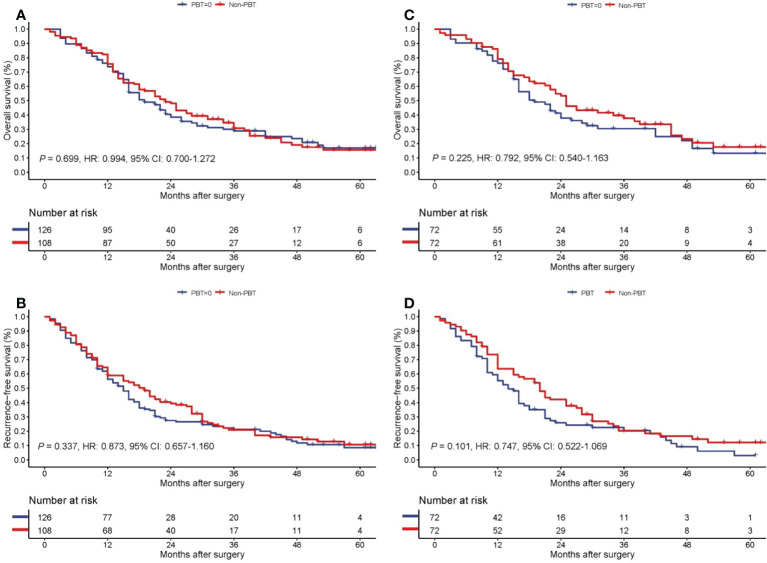
Kaplan−Meier curves of overall survival **(A)** and recurrence-free survival **(B)** for the PBT and non-PBT groups among patients with non-early stage (8th AJCC stage II-IV) pCCA treated with curative resection before PSM. Kaplan−Meier curves of overall survival **(C)** and recurrence-free survival **(D)** for the PBT and non-PBT groups among patients with non-early stage (8th AJCC stage II-IV) pCCA treated with curative resection after PSM. CI, confidence interval; HR, hazard ratio; PBT, perioperative blood transfusion; PSM, propensity score matching.

On follow-up, before PSM, the 5-year OS rates for patients with non-early stage pCCA treated with curative resection were 15.6% in the PBT group and 17.0% in the non-PBT group, respectively, while the 5-year RFS rates were 10.6% in the PBT group and 8.5% in the non-PBT group, respectively. After PSM, the 5-year OS rates for patients with non-early stage pCCA treated with curative resection were 17.7% in the PBT group and 13.3% in the non-PBT group, respectively, while the 5-year RFS rates were 12.1% in the PBT group and 3.0% in the non-PBT group, respectively (**Supplement Table 3**). Both before and after PSM, Kaplan–Meier curves revealed in patients with non-early stage pCCA, there were no significant differences between the PBT and non-PBT groups in OS and RFS (**Figure 3**).

## Discussion

PBT has been shown to be associate with perioperative safety of patients with hepatobiliary diseases. However, in some hepatobiliary diseases, such as hepatocellular carcinoma and colorectal liver metastases, immunomodulation brought on by PBT has been shown to associate with cancer recurrence (18–23). There have been very few studies reported on the association of PBT with long-term survival in pCCA patients. Radical resection of pCCA requires bile duct resection and reconstruction, hepatectomy, perihilar dissection, vascular resection and reconstruction if necessary, and it is a more complex, demanding, and high risk operation than resection for hepatocellular carcinoma or colorectal liver metastases. As a consequence, radical resection of pCCA has a greater need for PBT.

The association between PBT and long-term survival following resection for pCCA, to our knowledge, has only been studied in three previously published studies (8, 24, 25). Liu et al. observed a significant association between PBT and poor survival in 40 patients who underwent surgical resection for pCCA. However, blood transfusion could not be identified as an independent predictor in multivariate analysis of this study (24). In contrast, Young et al. demonstrated through multivariate analysis that PBT was a significant independent predictor of poor survival following surgery in a study of 83 patients with pCCA (25). Similarly, Kimura et al. retrospectively analysed the clinical data of 66 patients with pCCA who underwent surgical resection and found PBT to be an independent risk factor for poor OS and disease-free survival (8). The controversial results of the above three studies may well be due to differences in patient baseline characteristics, timings of the studies, tumor stagings and surgery types. However, in our opinion, the differences may be associated more with small sample sizes and selection biases of the studies. First, all these three studies had sample sizes of less than 100 patients coming from a single institution. The validity of the results of these studies could be improved by expanding the sample size and enrolling patients from multicenters. Second, as conducting a randomized controlled trial for PBT is not feasible due to ethical issues, PSM analysis can be used to minimize selection bias when randomized controlled studies cannot be carried out (26) in the same way as studies investigating the association between PBT and long-term survival of hepatocellular carcinoma patients using PSM analyses (11, 12).

To our knowledge, our study is the first study using PSM analysis and a multicenter database to investigate the impact of PBT on OS and RFS in patients with different stages of pCCA treated with curative resection. Of the 302 pCCA patients from three institutions included in this study, univariable analysis indicated that PBT did not adversely affect long-term survival of pCCA patients treated with curative resection. Two commonly used tumour staging systems or classifications were evaluated at the outset of this study, including the 8th AJCC staging system and the Bismuth classification to divide these patients into an early stage group and a non-early stage group to study the long-term survival of patients with different stagings of pCCA. The Bismuth classification was more relevant for choosing surgical procedures rather than classifying the degrees of tumor invasion. To better reflect the extent and location of tumor invasion, this study chose the 8th AJCC staging to group these patients. After grouping, the PBT rate of patients in the early stage group was significantly lower than that in the non-early stage group (32.4% vs. 46.2%). On long-term survival analysis, multivariable Cox regression analysis showed that PBT was independently associated with decreased OS and RFS rates in pCCA patients in the early-stage group treated with curative resection. However, in patients with non-early stage pCCA treated with curative resection, univariable analysis suggested that PBT had no significant effect on OS and RFS.

These exciting and interesting results can be explained by the conclusions drawn from the following reported studies. Blood transfusion has been well documented to increase immunosuppression in the host to promote cancer recurrence and metastasis. Blood transfusion in basic and clinical studie ahs been shown to decrease host immunity by reducing natural killer cell activity and cytotoxic T-cell function, increase suppressor T-cell activity, and decrease helper/suppressor (T4/T8) lymphocyte ratios (27, 28). In addition, normal physiological ageing and metabolic processes result in leaching of biologically active substances from cells into stored blood products. These leached bioactive substances have immunomodulatory effects that promote cell growth and angiogenesis and may therefore have a direct effect on tumor growth (29). The immunosuppressive impact of blood transfusion may therefore have a significant influence on recurrence of malignant tumors. A recent study by Goeppert et al. indicated that presence of both intratumoral T and B cells to be associated with prolonged survival in patients with cholangiocarcinoma and that prognosis was associated with inflammation (30). These findings provide a strong foundation in understanding the biological significance of inflammatory infiltrates in cholangiocarcinoma, as well as for further functional and clinical investigations on regulation of inflammatory responses in cholangiocarcinoma patients (30). Although immunosuppression may influence recurrence and survival in cholangiocarcinoma patients, the deleterious consequences of blood transfusion on host immunity remain unknown.

In our study, all patients were staged using the 8th AJCC staging system, and patients with early stage disease had tumors confined to the bile ducts. On the other hand, for individuals with non-early stage disease, their tumors had exhibited at least one of the following characteristics: invasion into surrounding adipose tissues, invasion into adjacent liver, invasion into one (or more) portal vein branches hepatic artery/common hepatic artery, lymph node invasion, and distant metastases. We hypothesize that the difference between the impact of PBT on prognosis of patients with early stage and non-early stage pCCA are the results of the difference in biological behaviors of the tumors in the 2 groups. PBT had detrimental effects on prognosis of patients with early stage disease, but its impact on prognosis of patients with more advanced diseases was obscured by the invasive and/or metastatic behavior of the tumors.

For pCCA patients who received PBT, the effects of postoperative adjuvant therapy remain to be further studied, as such a treatment way improve long-term survival. At present, immune checkpoint inhibitors have achieved remarkable results in biliary tract cancer, and some immune checkpoint inhibitors have achieved breakthroughs in clinical studies (clinical trial information: NCT03875235 and NCT03875235) (31). Since PBT could lead to immunosuppression in tumor patients who underwent radical surgery, it is worth studying whether such patients should receive adjuvant immunotherapy after surgery.

This study has several limitations. First, this retrospective study has its inherent defects, PSM analysis was used in this study to minimize selection bias. Second, there was only a small sample size of patients with early stage pCCA. However, as pCCA is a highly malignant tumor and it has no specific symptoms in the early stages, most patients in this study were already in the non-early stage at diagnosis. Patients enrolled in this study were much higher than those in other studies which investigated the association between long-term survival of pCCA patients with PBT. Third, patient selection and surgical procedures were not standardized among the three institutions in this study. For a multicenter study, such a bias cannot be completely be avoided. Despite this, the surgery was all performed by surgeons with rich experience in hepatobiliary surgery.

In conclusion, PBT was demonstrated in this study to be independently associated with worse long-term survival in patients with early stage pCCA treated with curative resection, but not in patients with non-early stage diseases. To improve the long-term survival of pCCA patients treated with curative resection, particularly those with early stage disease, PBT should be avoided if technically possible.

## Data availability statement

The raw data supporting the conclusions of this article will be made available by the authors, without undue reservation.

## Ethics statement

This study complied with the Declaration of Helsinki and was approved by the Ethics Committees of the 3 participating hospitals. The patients/participants provided their written informed consent to participate in this study.

## Author contributions

Conception, Z-YC, Z-PL; Study design, Z-PL, Z-JC, H-SD, S-YZ, D-CZ, Z-YC, S-QD; Administrative support, Z-YC, S-QD; Data collection and acquisition, S-YZ, J-HZ, X-YC, TY, X-CL, J-JC, JB, YJ; Data analysis, Z-PL, W-YC, Z-RW, Y-QZ; Manuscript preparation, Z-PL, Z-JC, H-SD; Critical revision, Z-YC, S-QD, WL; Final approval of manuscript, All authors.

## Funding

This work was supported in part by the National Natural Science Foundation of China (No. 81874211) and Chongqing Technology Innovation and Application Development Special Key Project (No. CSTC2021jscx-gksb-N0009).

## Conflict of interest

The authors declare that the research was conducted in the absence of any commercial or financial relationships that could be construed as a potential conflict of interest.

## Publisher’s note

All claims expressed in this article are solely those of the authors and do not necessarily represent those of their affiliated organizations, or those of the publisher, the editors and the reviewers. Any product that may be evaluated in this article, or claim that may be made by its manufacturer, is not guaranteed or endorsed by the publisher.

## Abbreviations

AJCC, American Joint Committee on Cancer; ALB, albumin; ALT, alanine aminotransferase; ASA, American Society of Anesthesiologists; AST, aspartate transaminase; CA 19-9, carbohydrate antigen 19-9; CI, confidence interval; HGB, haemoglobin; HR, hazard ratio; INR, international normalized ratio; IQRs, interquartile ranges; LN, lymph node; PBT, perioperative blood transfusion; pCCA, perihilar cholangiocarcinoma; PRBCs, packed red blood cells; PSM, propensity score matching; OR, odds ratio; OS, overall survival; RFS, recurrence-free survival; SDs, standard deviations.
