# Supplementary material for: Impact of perioperative blood transfusion on long-term survival in patients with different stages of perihilar cholangiocarcinoma treated with curative resection: A multicentre propensity score matching study
Source: Front Oncol. 2022 Oct 31;12:1059581. doi: 10.3389/fonc.2022.1059581 (PMC9660252; doi:10.3389/fonc.2022.1059581)
Supplement: Supplementary file 1 [file DataSheet_1.docx]

**Supplementary Material**

**Supplement Figure 1.** Patient selection flowchart. PBT, perioperative blood transfusion; pCCA, perihilar cholangiocarcinoma.


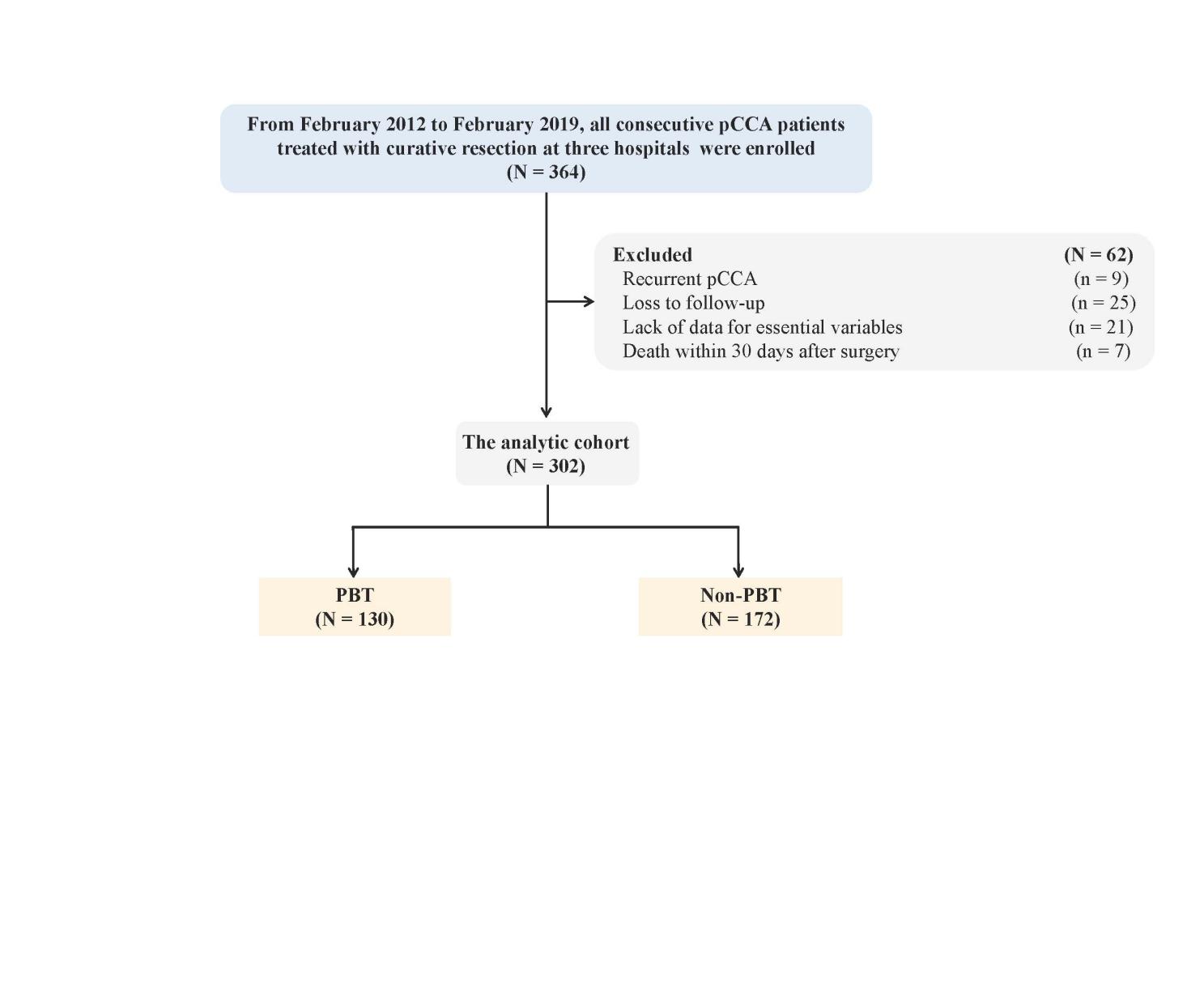


**Supplement Table 1.** Survival outcomes of the PBT and non-PBT groups among all pCCA patients treated with curative resection.

| Survival outcomes | Before PSM | | | After PSM | | |
| --- | --- | --- | --- | --- | --- | --- |
|  | PBT (n=130) | Non-PBT (n=172) | *P* value | PBT (n=90) | Non-PBT (n=90) | *P* value |
| Follow-up duration, months ^a^ | 23.0 (13.0, 36.0) | 22.0 (14.0, 41.8) | 0.632 | 25.0 (14.8, 39.3) | 23.5 (14.8, 40.3) | 0.265 |
| Death during follow-up | 96 (73.8) | 107 (62.2) | 0.003 | 64 (71.1) | 58 (64.4) | 0.339 |
| Recurrence during follow-up | 107 (82.3) | 130 (75.6) | 0.159 | 75 (83.3) | 69 (76.7) | 0.264 |
| OS, months ^b^ | 25.0 (21.0-29.0) | 28.0 (22.5-33.5) | 0.106 | 27.0 (20.8-33.2) | 29.0 (23.4-34.6) | 0.631 |
| 1-year OS, % | 79.1 | 80.2 |  | 86.6 | 83.3 |  |
| 3-year OS, % | 33.3 | 41.7 |  | 38.6 | 38.0 |  |
| 5-year OS, % | 18.9 | 29.4 |  | 22.7 | 27.8 |  |
| RFS, months ^b^ | 20.0 (16.0-24.1) | 18.0 (15.1-20.9) | 0.281 | 22.0 (15.0-29.0) | 20.0 (15.6-24.4) | 0.817 |
| 1-year RFS, % | 63.6 | 65.1 |  | 69.8 | 65.6 |  |
| 3-year RFS, % | 22.4 | 30.3 |  | 25.6 | 30.8 |  |
| 5-year RFS, % | 10.6 | 19.5 |  | 11.4 | 18.0 |  |

**Note,** ^a^ Values are shown as median (interquartile range); ^b^ Values are shown as median and 95% confidence interval.

**Abbreviations,** OS, overall survival; pCCA, perihilar cholangiocarcinoma; PBT, perioperative blood transfusion; PSM, propensity score matching; RFS, recurrence-free survival.

**Supplement Table 2.** Survival outcomes of the PBT and non-PBT groups among patients with early stage (8th AJCC stage I) pCCA treated with curative resection.

| Survival outcomes | Before PSM | | | After PSM | | |
| --- | --- | --- | --- | --- | --- | --- |
|  | PBT (n=22) | Non-PBT (n=46) | *P* value | PBT (n=18) | Non-PBT (n=18) | *P* value |
| Follow-up duration, months ^a^ | 29.0 (17.0, 40.0) | 40.5 (30.0, 61.3) | 0.020 | 28.5 (21.5, 37.5) | 37.5 (27.8, 60.0) | 0.141 |
| Death during follow-up | 14 (63.6) | 15 (32.6) | 0.016 | 11 (61.6) | 4 (22.2) | 0.018 |
| Recurrence during follow-up | 18 (81.8) | 25 (54.3) | 0.028 | 12 (66.7) | 6 (33.3) | 0.046 |
| OS, months ^b^ | 31.0 (17.7-44.3) | - | 0.005 | 36.0 (28.2-43.8) | - | 0.013 |
| 1-year OS, % | 95.5 | 97.8 |  | 94.4 | 100.0 |  |
| 3-year OS, % | 43.6 | 75.8 |  | 46.3 | 80.7 |  |
| 5-year OS, % | 32.6 | 62.2 |  | 20.6 | 72.6 |  |
| RFS, months ^b^ | 24.0 (5.1-42.9) | 59.0 (19.0-99.0) | 0.006 | 29.0 (16.2-41.8) | - | 0.039 |
| 1-year RFS, % | 86.4 | 89.1 |  | 94.4 | 94.4 |  |
| 3-year RFS, % | 29.8 | 55.4 |  | 23.0 | 60.7 |  |
| 5-year RFS, % | 13.2 | 47.9 |  | 23.0 | 60.7 |  |

**Note,** ^a^ Values are shown as median (interquartile range); ^b^ Values are shown as median and 95% confidence interval.

**Abbreviations,** AJCC, American Joint Committee on Cancer; OS, overall survival; pCCA, perihilar cholangiocarcinoma; PBT, perioperative blood transfusion; PSM, propensity score matching; RFS, recurrence-free survival.

**Supplement Table 3.** Survival outcomes of the PBT and non-PBT groups among patients with non-early stage (8th AJCC stage II-IV) pCCA treated with curative resection.

| Survival outcomes | Before PSM | | | After PSM | | |
| --- | --- | --- | --- | --- | --- | --- |
|  | PBT (n=108) | Non-PBT (n=126) | *P* value | PBT (n=72) | Non-PBT (n=72) | *P* value |
| Follow-up duration, months ^a^ | 22.0 (17.0, 35.8) | 18.0 (11.8, 29.3) | 0.281 | 25.0 (13.3, 37.5) | 18.0 (12.3, 29.0) | 0.106 |
| Death during follow-up | 82 (75.9) | 92 (72.0) | 0.611 | 51 (70.8) | 54 (75.0) | 0.574 |
| Recurrence during follow-up | 89 (82.4) | 105 (83.3) | 0.851 | 60 (83.3) | 62 (86.1) | 0.643 |
| OS, months ^b^ | 23.0 (18.8-27.2) | 19.0 (14.6-23.3) | 0.699 | 25.0 (21.0-24.3) | 19.0 (13.7-24.3) | 0.225 |
| 1-year OS, % | 75.8 | 73.8 |  | 79.0 | 76.2 |  |
| 3-year OS, % | 30.7 | 28.9 |  | 37.7 | 30.5 |  |
| 5-year OS, % | 15.6 | 17.0 |  | 17.7 | 13.3 |  |
| RFS, months ^b^ | 19.0 (14.6-23.5) | 15.0 (12.5-17.5) | 0.337 | 20.0 (16.4-23.6) | 14.0 (11.1-16.9) | 0.101 |
| 1-year RFS, % | 58.9 | 56.3 |  | 63.6 | 59.5 |  |
| 3-year RFS, % | 21.0 | 21.2 |  | 20.2 | 20.5 |  |
| 5-year RFS, % | 10.6 | 8.5 |  | 12.1 | 3.0 |  |

**Note,** ^a^ Values are shown as median (interquartile range); ^b^ Values are shown as median and 95% confidence interval.

**Abbreviations,** AJCC, American Joint Committee on Cancer; OS, overall survival; pCCA, perihilar cholangiocarcinoma; PBT, perioperative blood transfusion; PSM, propensity score matching; RFS, recurrence-free survival.
